# Lumbar spinal canal dimensions measured intraoperatively after decompression are not properly rendered on early postoperative MRI

**DOI:** 10.1007/s00701-016-2777-5

**Published:** 2016-03-23

**Authors:** Catharina Schenck, Job van Susante, Maarten van Gorp, Ruben Belder, Carmen Vleggeert-Lankamp

**Affiliations:** Department of Neurosurgery, Leiden University Medical Centre, Albinusdreef 2, 2333 ZA Leiden, The Netherlands; Department of Orthopaedics, Rijnstate Hospital, Arnhem, The Netherlands; Department of Radiology, Rijnstate Hospital, Arnhem, The Netherlands

**Keywords:** Lumbar spinal stenosis, Surgical decompression, Prone and supine MRI, Intraoperative versus postoperative dimensions of the spinal canal, Direct intraoperative measurement of spinal canal, Treatment outcome

## Abstract

**Background:**

In cases of lumbar spinal stenosis (LSS) treated with surgical decompression, a postoperative magnetic resonance imaging (MRI) is sometimes required. In the experience of the investigators of this study, the obtained decompression observed on early postoperative MRI tends to be disappointing compared to the decompression achieved intraoperatively. This raises the question of whether the early postoperative MRI, performed after lumbar decompression, is a fair representation of the ‘real’ decompression. This study investigated the correlation between intraoperative and postoperative measurements of the lumbar spinal canal.

**Method:**

Twenty patients with LSS underwent surgical decompression on a single level. The orthopaedic surgeon performed direct intraoperative measurements of width, length and height of the spinal canal. Preoperative supine MR images and postoperative prone and supine MR images were acquired. Two radiologists (R.B. and M.G.) measured width, length and height of the spinal canal on the preoperative and postoperative MRIs. Intraoperative measurements were compared to measurements on postoperative MRI in prone position (thus reproducing the intraoperative situation) to avoid positioning bias. Preoperative and postoperative measurements on MR images were also compared. In addition to this, postoperative measurements on supine and prone MR images were also compared.

**Results:**

Interobserver reliability for MRI measurements by both radiologists was generally excellent (intraclass correlation coefficients ≥0.71). The postoperative spinal canal dimensions improved on both prone and supine MRI compared to the preoperative imaging (*P* < 0.05). Intraoperatively measured dimensions demonstrated a significantly greater height (difference 2.8 ± 3.3 [R.B.] and 1.9 ± 3.7 [M.G.]) and greater width (difference 2.1 ± 3.2 [R.B.] and 2.5 ± 2.7 [M.G.]) compared to postoperative MRI in the prone position (*P* < 0.05). Postoperative dural sac height was greater on the supine MRI compared to the prone MRI (*P* < 0.05).

**Conclusions:**

Surgical decompression of the spinal canal effectively decreases the compression of the dural sac. However, early postoperative MRI after lumbar decompression does not adequately represent the decompression achieved intraoperatively.

## Introduction

Intermittent neurogenic claudication (INC) caused by severe lumbar spinal stenosis (LSS) is common in the elderly [6, 7, 16]. Upon progressive narrowing of the lumbar spinal canal, patients start to develop the typical symptoms due to compression of the roots of the cauda equina: leg pain (frequently in both legs), exacerbated by walking, prolonged standing or lumbar extension, and sometimes associated back pain [2–4, 16, 17]. Surgical treatment is considered to be superior to non-surgical treatment [13, 14], but patient satisfaction after treatment is relatively low—about 70 % in large studies [1, 13, 14]. This disappointing outcome was believed to be caused by the destructive nature of bony decompression [5, 18, 19], and the ongoing degeneration of the lumbar spine and the facet joints, in particular, that generally accompanies spinal stenosis.

In patients in which a surgical decompression was performed in order to relieve the symptoms, and in which the results are not satisfactory with respect to the leg pain, a postoperative magnetic resonance imaging (MRI) is performed every now and then in search of an explanation of the persisting complaints. In the experience of the investigators of this study, the obtained decompression observed on early postoperative MRI tends to be disappointing when comparing to the surgical intraoperative view. This raises the question of whether the early postoperative MRI, performed after lumbar decompression, is a fair representation of the ‘real’ decompression and whether the MR images correlate to the postoperative clinical condition of the patient.

Two studies were retrieved that reported on early routine MRI studies after lumbar decompression surgery [9, 12]. In a study performed by Schubert et al. [12], 28 patients who underwent bilateral interlaminar fenestration on multiple levels had undergone a postoperative MRI within 72 h after surgery. At the time of the postoperative MRI, none of the patients had reported new paresis, hypesthesia, bladder or bowel dysfunction, or progressive leg or back pain. Patients reported significant improvement in symptom severity scores early after surgery. However, in as much as 66.7 % of patients, at least one lumbar level was still found to be moderately to severely compressed on the postoperative MR images. A study performed by Oba et al. [9] included 83 patients who underwent various forms of posterior lumbar decompression surgery. Postoperative MR images demonstrated that the mean cross-sectional area between the early (within 1 week) and late (more than 1 month) postoperative phases increased significantly, indicating that direct postoperative MRI is not representative for long-term results.

While there is some literature on the evolution of the dural sac and spinal canal measurements before and after lumbar decompression surgery, there is no literature on the correlation between the intraoperative measurement of the dimensions of the decompressed lumbar canal and postoperative dimensions on MRI scan. A better understanding of the relationship between intraoperative and postoperative measurements will allow for a better evaluation of the postoperative MR images early after surgery in case they are performed. The purpose of this study was therefore to investigate the correlation between preoperative, intraoperative and postoperative measurements of the symptomatic and subsequently decompressed level of the lumbar spinal canal on MRI.

## Materials and methods

### Patient population

Institutional review board agreement was obtained for this prospective cohort study (LTC number 876/310812). The number of patients intended for inclusion was 22. Informed consent was obtained for each patient included in the study. Included patients satisfied the following inclusion criteria: neurogenic claudication of degenerative origin at the levels of L3-S1 requiring single-level lumbar decompression, age between 30 and 80 years, male or female and no history of previous lumbar spine surgery at the affected spinal level. None of the patients presented lumbar instability.

### Surgical intervention

Patients underwent surgery in a prone position, in which they were placed on three gel cylinders placed under the pelvis, thorax and ankles. The level of incision was confirmed by fluoroscopy. A midline incision was made, after which the muscles were detached from the spinous processes. The interspinous ligament was removed, as well as a small rim of the spinous processes. A flavectomy was performed with bilateral opening of the lateral recess using a Kerrison punch. A bony decompression was performed by removing the caudal rim of the rostral lamina and, if needed, a minimal medial facetectomy was performed. If necessary, the rostral rim of the caudal lamina was removed.

An intraoperative myelogram was made after the decompression to ascertain adequate decompression and expansion of the dural sac. To that end, 10 ml of Omnipaque™ (300 mg I/ml) was introduced via the midline through a syringe into the intradural space. Before removing the needle, the hole was surfaced with 2 ml of Tissucol® in order to prevent the contrast from leaking and thereby interfering with the myelogram. After injecting the contrast fluid, a lateral fluoroscopy scan was performed of the area of interest. At the end of each procedure the dural sac was decompressed satisfactorily.

Afterwards, the wound was closed and no drain was left in place. Patients were mobilised on the 1st day after surgery with the help of a physiotherapist.

### MRI protocol

MRI scans were performed using standardised protocols tailored to a 1.5-T Siemens Magnetom Avanto scanner (Siemens, Erlangen, Germany). Sagittal T1 TSE and sagittal and axial T2 TSE images of the lumbar spine were acquired. Slice thickness was 3.5 mm for sagittal series and 4 mm for axial series.

**Fig. 1:**
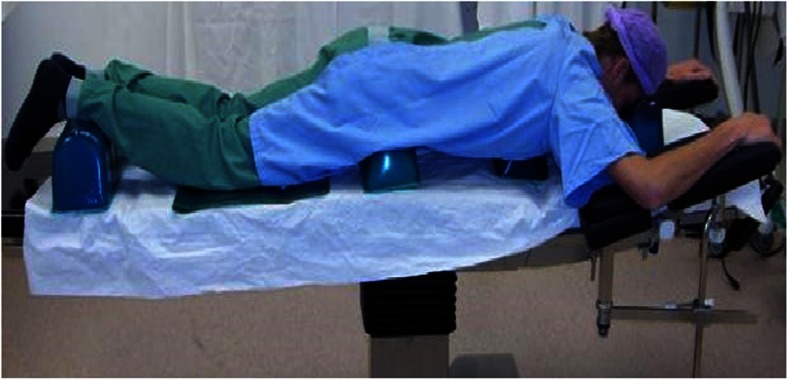
Simulation of the prone position in which patients were operated on and in which they underwent postoperative MRI

Preoperative MRI scans in the supine position were available for all patients. Postoperative MRI scans were made in both the supine and the prone position and were performed on the day following the surgery. Initially all patients only underwent one postoperative MRI in prone position. After a few surgeries it was decided that patients should also undergo a supine postoperative MRI. Therefore, nine patients in our patient population had both types of postoperative MRI. The prone position was chosen to reproduce the positioning of the patient during the surgical intervention (Fig. 1). In the prone position, three gel cylinders were placed under the pelvis and the thorax in order to obtain flexion of the lumbar spine. Since MR images are not usually taken in the prone position, feasibility and reproducibility of the procedure was tested beforehand on volunteers. In addition, MRI scans were calibrated against a reference marker with known size placed above the wound to ensure accurate and valid correlation between intraoperative direct measurements and postoperative dural sac measurements on MRI. The supine postoperative MRI was performed in order to compare the postoperative size of the canal on supine and prone MRI.

### MRI evaluation

Two neuroradiologists (M.G. and R.B.) independently evaluated all MR images according to a predefined protocol. The readers were not provided any clinical information and were not involved in the selection or care of the included patients. Observer experience in reading spine MRIs was 7 years post-residency and 6 years as a resident.

**Fig. 2:**
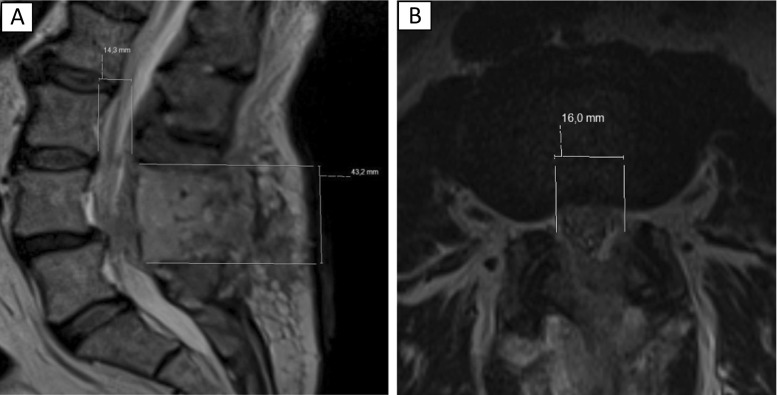
**a** Measurements of height (14.3 mm) and length (43.2 mm) on MRI. **b** Measurement of width (16.0 mm) on MRI

The neuroradiologists conferred and agreed on the relevant T2 axial and sagittal MRI slices before performing measurements on these predetermined slices. Subsequent measurements by the neuroradiologists were performed independently. They measured height, width and length in the spinal canal, which were defined as (Fig. 2):

- Height: size of the dural sac (distance in millimetres) from posterior rim of vertebral body to anterior rim of the lamina on an axial slice at the level of the intervertebral disc.
- Width: size of the dural sac (distance in millimetres) from the lateral border of the dural sac adjacent to the medial rim of the left pedicle with (the remainders of) the ligamentum flavum to the lateral border of the dural sac adjacent to the medial rim of the right pedicle with (the remainder of) the ligamentum flavum on an axial slice.
- Length: distance (millimetres) from the inferior border of the superior (partially removed) lamina to the superior border of the inferior (partially removed) lamina, on a sagittal slice, in the midline of the vertebral body.

Width and height were measured both preoperative and postoperatively. Length was only measured postoperatively, since the length of the interlaminar spinal canal was increased by definition, using the decompressive technique of the lumbar spinal canal that was described above.

### Intraoperative measurements by the orthopaedic surgeon

**Fig. 3:**
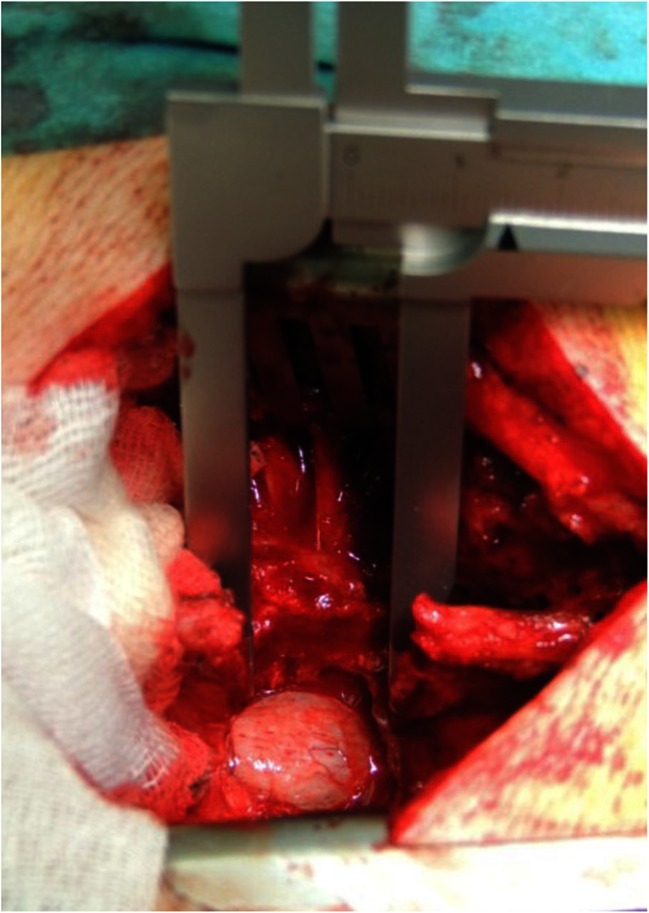
Intraoperative measurement of the length of the decompressed area using a caliper

After the surgeon (J.S.) had completed the surgical decompression of the affected level, direct intraoperative measurements of the spinal canal were performed using a caliper (Fig. 3). The caliper was placed between the rims of the bilateral facets to measure the width of the canal, and between the superior and inferior lamina, in the midline, in order to measure the length of the decompressed level. An instrument to measure the depth of the canal was placed on the vertebra, at the level of the intervertebral disc and the distance to the posterior border of the caudal sac was measured in order to obtain the height of the canal.

### Intraoperative myelography

**Fig. 4:**
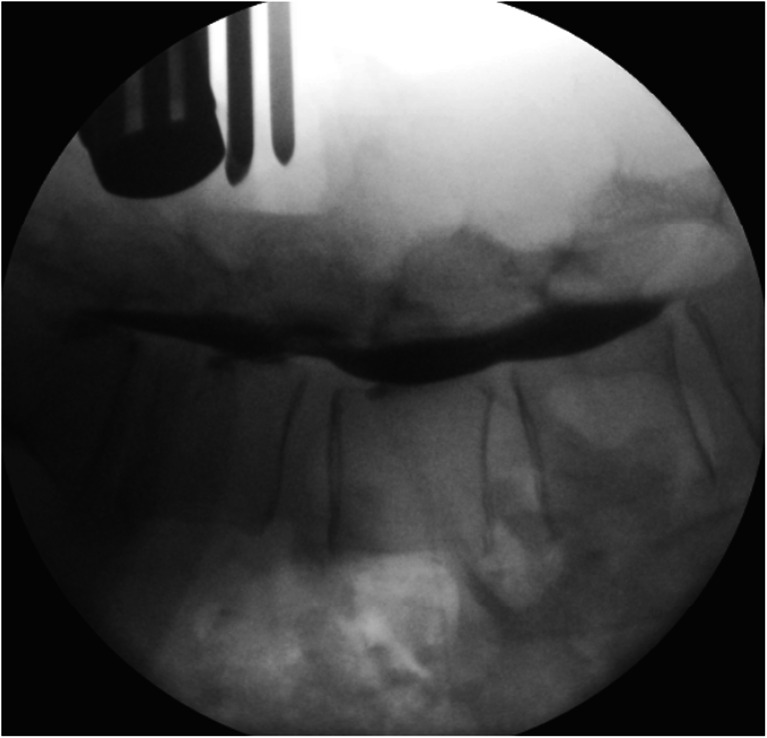
Intraoperative myelography. Note the instrument handle with known diameter (25 mm) held in the operative field at time of fluoroscopy for calibration purposes of the measurements

Additional perioperative information was obtained via myelography (Fig. 4). After the injection of contrast, lateral fluoroscopic images were obtained. The height of the canal was obtained by measuring the contrast column from the posterior rim of the vertebra to the ventral rim of the lamina at the level of the intervertebral disc. Measurements on myelography were calibrated against the known diameter (25 mm) of one of the instruments used and held (Fig. 4) in the operative field at time of fluoroscopy.

### Outcomes

The clinical outcome of patients was assessed by means of the Roland Disability Questionnaire for Sciatica (RDQS, scores ranging from 0 to 23, with higher scores indicating worse functional status), the 100-mm visual-analogue scale (VAS) for leg and back pain (with 0 representing no pain and 100 the worst pain ever experienced), and a 7-point Likert self-rating scale of global perceived recovery, with answers ranging from completely recovered (1) to much worse (7) [10, 11]. Outcome measures were assessed at baseline and at 8 weeks after surgery. Patients were blinded to results of the earlier assessment. Subsequently, outcome scores were correlated to the difference in preoperative and postoperative spinal canal dimensions.

### Statistical analysis

The intraclass correlation coefficient was used to determine the interobserver agreements for both neuroradiologists on MRI measurements [15]. While no absolute definitions have been accepted for the interpretation of interobserver agreements, we used guidelines proposed by Landis and Koch for interpretation [8]. Values from 0.00 to 0.20 indicated slight reliability; 0.21–0.40, fair reliability; 0.41–0.60, moderate reliability; 0.61–0.80, substantial reliability; 0.81–1.00, excellent reliability. Differences between preoperative and postoperative clinical and MRI parameters were assessed using paired *t*-tests for continuous data. Correlations between clinical and MRI parameters were evaluated with Spearman correlation tests. Statistical significance was defined as *P* <0.05. All analyses were performed using SPSS statistics version 21.

## Results

### Patient population

Two of the 22 primarily included patients had to be excluded. In one of the patients, a dural tear was experienced during the surgical intervention, which made the measurements in this patient not fully comparable with the other patients. In another patient, the myelogram that was made during surgery for the sake of this study revealed compression on an additional level. To that end, this extra level was also decompressed, and the patient was excluded from this study, since outcome parameters could be influenced by the operation on an additional level. Nine patients underwent decompression at level L3-L4, 11 patients underwent decompression at level L4-L5.

### MRI interobserver agreement

Interobserver agreements on the measurements of width and height of the affected level on the preoperative MRIs by the neuroradiologists were excellent. Likewise, interobserver agreements on all measurements performed on postoperative supine MRIs were excellent. Interobserver agreements on measurements performed on postoperative prone MRIs were excellent for length and width and substantial for height (Table 1).

**Table 1:** Interobserver agreements for both neuroradiologists on MRI measurements

		Interobserver agreements	
		Supine MRI	Prone MRI
Preoperative measurements	Length	n.a.	n.a.
	Width	0.89	n.a.
	Height	0.82	n.a.
Postoperative measurements	Length	0.99	0.98
	Width	0.96	0.92
	Height	0.90	0.71

Statistics: intraclass correlation coefficient

*n.a.* not available

### MRI measurements

Comparing the neuroradiologists’ measurements of width and height of the spinal canal performed on preoperative and postoperative MRIs in the supine position, a significant expansion of the dural sac was observed. The mean width increased from 9 ± 1.6 mm to 12.8 ± 2.3 mm with a mean improvement of 4.1 mm (M.G.) and from 9 ± 1.9 mm to 12.8 ± 2.3 mm with a mean improvement of 3.9 mm (R.B.). The mean height increased from 11.4 ± 2.3 mm to 16.1 ± 2.3 mm with a mean improvement of 4.4 mm (M.G.) and from 11.7 ± 2.1 mm to 15.7 ± 2.8 mm with a mean improvement of 3.2 mm (R.B.). A comparable increase was observed in comparing measurements of width and height on preoperative supine MRI and postoperative prone MRI (Table 2).

**Table 2:** Increase in height and width of the dural sac after the surgery: comparison of preoperative supine MRI with postoperative supine and prone MRI

		Preoperative supine MRI (*n* = 20)	Postoperative supine MRI (*n* = 9)	Postoperative prone MRI (*n* = 20)
Radiologist 1 (R.B.)	Mean height in mm (±SD)	11.7 (±2.1)	15.7 (±2.8) 3.2 (±1.5)^a^ <0.001^b^	12.8 (±2.8) 1.1 (±2.5)^a^ 0.065^b^
	Mean width in mm (±SD)	9.0 (±1.9)	12.8 (±2.3) 3.9 (±3.2) ^a^ 0.006^b^	12.5 (±3.4) 3.5 (±3.3)^a^ <0.001^b^
Radiologist 2 (M.G.)	Mean height in mm (±SD)	11.4 (±2.3)	16.1 (±2.3) 4.4 (±2.2)^a^ <0.001^b^	13.7 (±3.0) 2.3 (±3.0)^a^ 0.004^b^
	Mean width in mm (±SD)	9.0 (±1.6)	12.8 (±2.3) 4.1 (±2.3)^a^ <0.001^b^	12.1 (±2.8) 3.1 (±2.7)^a^ <0.001^b^

Statistics: paired *t*-test

*n* number of patients, *mm* millimetres, *SD* standard deviation

^a^Mean increase compared to preoperative MRI (mean of paired differences)

^b^ *P* value for comparison with preoperative MRI

### Intraoperative evaluation

The dimensions of the decompressed spinal canal measured by the orthopaedic surgeon during surgery were compared with the dimensions of the spinal canal measured by the radiologists on the calibrated postoperative MRIs (Table 3). MRI in the prone position was chosen instead of the supine position to avoid possible influence of position on the dimensions of the spinal canal. The intraoperatively measured heights and widths of the decompressed canal were significantly greater than the heights and widths measured on postoperative MR images. Between the intraoperative and the early postoperative situation, height decreased on average by 2.8 ± 3.3 mm (R.B.) and 1.9 ± 3.7 mm (M.G.), while width decreased by 2.1 ± 3.2 mm (R.B.) and 2.5 ± 2.7 mm (M.G.). Length was the only parameter which did not appear to be smaller postoperatively.

**Table 3:** Decrease in height and width of the dural sac in postoperative phase: intraoperative dimensions measured by orthopaedic surgeon compared to postoperative dimensions on prone MRI

	Intraoperative (*n* = 20)	Postoperative prone MRI (*n* = 20)	
	Orthopaedic surgeon	Radiologist 1 (R.B.)	Radiologist 2 (M.G.)
Mean height in mm (±SD)	15.6 (±2.8)	12.8 (±2.8) −2.8 (±3.3)^a^ <0.001^b^	13.7 (±3.0) −1.9 (±3.7)^a^ 0.032^b^
Mean width in mm (±SD)	14.6 (±1.4)	12.5 (±3.4) −2.1 (±3.2)^a^ 0.008^b^	12.1 (±2.8) −2.5 (±2.7)^a^ <0.001^b^
Mean length in mm (±SD)	17.7 (±5.9)	22.2 (±7.0) 4.5 (±3.3)^a^ <0.001^b^	22.2 (±7.1) 4.5 (±3.9)^a^ <0.001^b^

Statistics: paired *t*-test

*n* number of patients, *mm* millimetres, *SD* standard deviation

^a^Mean change compared to intraoperative situation (mean of paired differences)

^b^ *P* value for comparison with intraoperative measurements

The intraoperative myelogram was only evaluated for the height of the spinal canal because only lateral images were obtained. The mean height of the contrast column was significantly smaller than the height measured with caliper and on MRI (Table 4).

**Table 4:** Height measured on myelography compared to intraoperative and postoperative height

	Myelography (*n* = 20)	Intraoperative measurements by orthopaedic surgeon (*n* = 20)	Postoperative prone MRI (*n* = 20)	Postoperative supine MRI (*n* = 9)
Mean height in mm (±SD)	8.6 (±2.4)	15.6 (±2.8) 7.0 (±3.3)^a^ 0.000^b^	R.B.: 12.8 (±2.8) 4.2 (±3.6)^a^ 0.000^b^	R.B.: 15.7 (±2.8) 7.2 (±3.4)* 0.000^b^
			M.G.: 13.7 (±3.0) 5.1 (±4.3)^a^ 0.000^b^	M.G.: 16.1 (±2.3) 7.6 (±2.7)* 0.000^b^

Statistics: paired *t*-test

*n* number of patients, *mm* millimetres, *SD* standard deviation

^a^Mean difference compared to myelography (mean of paired differences)

^b^ *P* value for comparison with myelography

### Prone versus supine MRI

The dimensions of the spinal canal on postoperative supine MRI were compared to the dimensions of the spinal canal on postoperative prone MRI. The height of the spinal canal measured on prone MR images was significantly smaller than on regular supine MR images. The average difference was 2.22 ± 1.9 mm (R.B.) and 2.22 ± 2.1 mm (M.G.). The other parameters did not vary significantly according to position (Table 5).

**Table 5:** Comparison of dimensions of the dural sac on postoperative MRI according to position

		Postoperative supine MRI (*n* = 9)	Postoperative prone MRI (*n* = 20)	Mean difference in mm (±SD)	*P* value
Radiologist 1 (R.B.)	Mean height in mm (±SD)	15.7 (±2.8)	12.8 (±2.8)	−2.22 (±1.9)	0.007
	Mean width in mm (±SD)	12.8 (±2.3)	12.5 (±3.4)	−1.56 (±3.0)	0.154
	Mean length in mm (±SD)	23.0 (±8.9)	22.2 (±7.0)	1.11 (±1.8)	0.107
Radiologist 2 (M.G.)	Mean height in mm (±SD)	16.1 (±2.3)	13.7 (±3.0)	−2.22 (±2.1)	0.013
	Mean width in mm (±SD)	12.8 (±2.3)	12.1 (±2.8)	−1.56 (±2.8)	0.138
	Mean length in mm (±SD)	23.7 (±8.7)	22.2 (±7.1)	0.00 (±3.0)	1.000

Statistics: paired *t*-test

*n* number of patients, *mm* millimetres, *SD* standard deviation

### Outcome

The mean VAS scores improved significantly from 44.3 to 16.1 mm (leg pain) and from 52.7 to 26.8 mm (back pain) on a 100-mm scale (Table 6). The mean RDQS improved significantly from 13.5 to 5.8 (on a 23-point scale). Postoperative patient satisfaction (Likert) scores were good, on average 1.9 on a 7-point scale. In only six patients were the preoperative and postoperative dimensions in the supine position plus the corresponding preoperative and postoperative outcome scores available. The improvement score was obtained by subtracting the preoperative from the postoperative outcome scores for each individual patient. In this limited number of complete patient data, no significant correlation in improvement scores and increase in height or width of the lumbar spinal canal assessed on the prone MRI was established; nor in improvement scores and intraoperatively measured dimensions.

**Table 6:** Improvement in outcome scores

	Mean preoperative score (±SD)	Mean postoperative score (±SD)	*P* value
VAS (leg pain) *n* = 16	44.3 (±29.7)	16.1 (±17.4)	0.001
VAS (back pain) *n* = 16	52.7 (±26.5)	26.8 (±29.5)	0.006
RDQS *n* = 17^a^	13.5 (±5.2)	5.8 (±5.9)	0.000
Likert *n* = 17	n.a.	1.9 (±1.1)	n.a.

Statistics: paired *t*-test

*n.a.* not available, *n* number of patients, *SD* standard deviation

^a^Postoperative number of patients was 16

## Discussion

Comparison of the preoperative and postoperative dimensions of the lumbar spinal canal on MRI after interlaminar decompression showed a significant increase, suggesting that surgical intervention effectively decreases the compression of the dural sac seen in lumbar spinal stenosis.

However, the dimensions measured by the surgeon during operation after the decompression are significantly greater than those measured on postoperative MRI, even if this MRI is made just 1 day after the surgery in the same prone position in which the surgical decompression was performed. Therefore, early postoperative MRI after lumbar decompression does not adequately represent the actual condition of the lower spinal canal.

A possible explanation for disappointing results on early postoperative imaging could be postoperative swelling of the structures in the canal in combination with mild haematomas. This is in agreement with the study by Oba et al. [9], which showed a significant improvement in the dural sac cross-sectional area between postoperative MR images taken within a week after lumbar decompressive surgery and the postoperative MR images taken a month or more after surgery. The swelling that they propose as an explanation for their results, may well explain the difference between intraoperative and postoperative dimensions that we established.

In our study we corrected for changes in lumbar spinal canal morphology due to different postures between the intraoperative situation (prone) and the radiological situation (supine). This cannot, therefore, be used as an argument to explain the differences seen in intraoperative dimensions by the surgeon and postoperative dimensions on MRI.

It was established that the height of the dural sac was significantly smaller on prone MR images, suggesting that position does play a role in spinal canal morphology. This could be explained by increased lordosis and therefore increased compression of the dural sac when patients are lying in the prone position.

Measurements performed on the intraoperative myelogram demonstrated a mean height that was not in agreement with the dimensions measured by the orthopaedic surgeon using the caliper, nor with the postoperative MRIs. Therefore, we can conclude that a myelogram is not fit to judge the dimensions of the canal and that it should be reserved for those cases in which the surgeon wants to establish the severity of the stenosis by judging whether fluid can still circulate past the stenotic level.

In our study, we found significant overall improvement in the dimensions of the lumbar spinal canal and dural sac after surgery. The limited number of patients studied for which preoperative and postoperative scores as well as preoperative and postoperative measurements on supine MR images were available prevents us from drawing sound conclusions on correlations between canal dimensions and patient recovery. To that end, we have performed another study in which we compared clinical and radiological parameters in a large cohort (accepted for publication). The trend which was observed here—namely, that quantitative canal dimension parameters should not be regarded as predictors for clinical outcome—is confirmed in that other study.

## Conclusions

Early evaluation of decompression on postoperative MRI after decompressive surgery in patients with lumbar spinal canal stenosis is only of limited value in assessing the extent of the actual decompression. The surgeon’s impression of the size of the decompressed spinal canal is more informative. Future studies should aim at studying the dimensions of the lumbar spine in the late postoperative phase, to see whether the dimensions return to the extent measured intraoperatively.
